# Risk of adverse outcome of COVID-19 among patients in secure psychiatric services: observational cohort study

**DOI:** 10.1192/bjo.2020.169

**Published:** 2021-01-11

**Authors:** Natasa Basrak, Naoise Mulcrone, Sue Sharifuddin, Zeshan Ghumman, Nirvana Bechan, Enas Mohamed, Michael Murray, Hariharan Rajendran, Sean Gunnigle, Mark Nolan, Tim Quane, Masashi Terao, Tracey Hoare, Kevin Kirrane, Harry G. Kennedy, Mary Davoren

**Affiliations:** National Forensic Mental Health Service, Central Mental Hospital, Dublin, Ireland; National Forensic Mental Health Service, Central Mental Hospital, Dublin, Ireland; National Forensic Mental Health Service, Central Mental Hospital, Dublin, Ireland; National Forensic Mental Health Service, Central Mental Hospital, Dublin, Ireland; National Forensic Mental Health Service, Central Mental Hospital, Dublin, Ireland; National Forensic Mental Health Service, Central Mental Hospital, Dublin, Ireland; National Forensic Mental Health Service, Central Mental Hospital, Dublin, Ireland; National Forensic Mental Health Service, Central Mental Hospital, Dublin, Ireland; National Forensic Mental Health Service, Central Mental Hospital, Dublin, Ireland; National Forensic Mental Health Service, Central Mental Hospital, Dublin, Ireland; and DUNDRUM Centre for Forensic Excellence, Trinity College Dublin, Ireland; National Forensic Mental Health Service, Central Mental Hospital, Dublin, Ireland; National Forensic Mental Health Service, Central Mental Hospital, Dublin, Ireland; National Forensic Mental Health Service, Central Mental Hospital, Dublin, Ireland; National Forensic Mental Health Service, Central Mental Hospital, Dublin, Ireland; National Forensic Mental Health Service, Central Mental Hospital, Dublin; and DUNDRUM Centre for Forensic Excellence, Trinity College Dublin, Ireland; National Forensic Mental Health Service, Central Mental Hospital, Dublin; and DUNDRUM Centre for Forensic Excellence, Trinity College Dublin, Ireland

**Keywords:** COVID-19, schizophrenia, forensic mental health services, risk assessment, obesity

## Abstract

**Background:**

Secure forensic mental health services treat patients with high rates of treatment-resistant psychoses. High rates of obesity and medical comorbidities are common. Population-based studies have identified high-risk groups in the event of SARS-CoV-2 infection, including those with problems such as obesity, lung disease and immune-compromising conditions. Structured assessment tools exist to ascertain the risk of adverse outcome in the event of SARS-CoV-2 infection.

**Aims:**

To assess risk of adverse outcome in the event of SARS-CoV-2 infection in a complete population of forensic psychiatry patients using structured assessment tools.

**Method:**

All patients of a national forensic mental health service (*n* = 141) were rated for risk of adverse outcome in the event of SARS-CoV-2 infection, using two structured tools, the COVID-Age tool and the COVID-Risk tool.

**Results:**

We found high rates of relevant physical comorbidities. Mean chronological age was 45.5 years (s.d. = 11.4, median 44.1), mean score on the COVID-Age tool was 59.1 years (s.d. = 19.4, median 58.0), mean difference was 13.6 years (s.d. = 15.6), paired *t* = 10.9, d.f. = 140, *P* < 0.001. Three patients (2.1%) were chronologically over 70 years of age, compared with 43 (30.5%) with a COVID-Age over 70 (χ^2^ = 6.99, d.f. = 1, *P* = 0.008, Fisher's exact test *P* = 0.027).

**Conclusions:**

Patients in secure forensic psychiatric services represent a high-risk group for adverse outcomes in the event of SARS-COV-2 infection. Population-based guidance on self-isolation and other precautions based on chronological age may not be sufficient. There is an urgent need for better physical health research and treatment in this group.

Patients with severe mental illness detained in secure forensic hospitals may be at increased risk of adverse outcomes in the event of infection with SARS-CoV-2. Early reports indicate not only that outbreaks can spread quickly within such settings but that adverse outcomes can occur in relatively young patients.^1^ Severe acute respiratory syndrome coronavirus-2 (SARS-CoV-2), the cause of the coronavirus disease 2019 (COVID-19), was first reported in Wuhan, China, in December 2019.^2^ By March 2020, the World Health Organization (WHO) had declared COVID-19 a pandemic. By September 2020 the virus had infected 28.6 million people, claiming more than 1 million lives. COVID-19 is associated with severe acute respiratory syndrome and the need for intensive care unit (ICU) admission.^3,4^ The mortality rate of COVID-19 is 3–4%, up to 40 times that of seasonal influenza.^5^ Transmission is person-to-person and up to 80% of infected individuals have either mild symptoms or are asymptomatic. Individuals can be infectious during the incubation and asymptomatic periods, adding to the difficulty in managing the pandemic.^6,7^ Statistical models estimated that, with no mitigation efforts, an estimated 40 million deaths would occur worldwide from COVID-19,^8^ resulting in the use of population-based measures to restrict person-to-person transmission.^9,10^

## Vulnerable groups and vulnerable settings

The clinical course of COVID-19 is highly variable.^3,11,12^ Risk factors associated with adverse outcome in the event of SARS-CoV-2 infection include increased age, obesity, chronic cardiac disease, diabetes, hypertension and other chronic medical conditions.^4,12–14^ Higher death rates from COVID-19 have been shown among groups with intellectual disability, possibly because people with intellectual disability having higher rates of medical conditions such as epilepsy, dysphagia and pneumonia.^15^ Individuals from Black and minority ethnic backgrounds are more likely to have a poorer outcome in the event of infection.^16^ Case fatality rates in individuals over 70 years of age range from 5 to 30%.^17,18^ Therefore, public health guidance in many jurisdictions recommends that healthy adults aged 70 or more should take special precautions, self-isolating or ‘cocooning’.

Environments such as care homes, which by definition have older populations including those who are medically vulnerable, have been disproportionately affected by adverse outcomes during the pandemic^19,20^ although the lack of systematic data available from these settings has impeded the monitoring of the rates of illness and outcomes among this vulnerable group.^21^ Owing to shared living situations, COVID-19 can spread rapidly in longer-term residential facilities^22^ and mortality from COVID-19 infections has been concentrated in longer-term residential or care home facilities.^23^

Secure forensic mental health services provide care and treatment to mentally disordered offenders, the vast majority of whom have major mental illness, most commonly treatment-resistant schizophrenia.^24–26^ Forensic services have a dual role, to treat mental illness and reduce violent recidivism.^24^ There are high rates of physical health comorbidities among this group, particularly obesity, hypertension and metabolic syndrome, and high rates of neurocognitive impairment.^27–30^ People with major mental illnesses are more likely to become infected with COVID-19 in the community and are more than twice as likely to have a severe outcome from COVID-19 infection than the general population.^31–33^ Outbreaks have occurred worldwide in psychiatric centres, including forensic hospitals, with fatal results.^1,2^ High transmission rates have been found within psychiatric units despite implementation of Centers for Disease Control and Prevention (CDC) guidelines.^34^ In federal and state prisons in the USA, the rate of infection is higher than in the general population.^35^ The length of stay in secure forensic hospitals can exceed 5 years for a significant proportion of patients.^26,36^ Therefore, patients in secure forensic hospitals, who have high levels of treatment-resistant mental illness, complicated by violence, neurocognitive impairment and complex physical health needs, may be a uniquely vulnerable group in the event of infection with SARS-CoV-2.

### COVID-Risk and COVID-Age assessment tools

Two scales to assess the risk of adverse outcome in the event of SARS-CoV-2 infection are available, the COVID-Risk assessment tool developed by the British Medical Association (BMA) and the COVID-Age assessment tool developed by the Association of Local Authority Medical Advisors (ALAMA).^37,38^

The COVID-Risk assessment tool lists vulnerabilities such as gender, age and various physical health conditions, and attributes a weighted score to each. The patient's total score is added together to place the patient into one of three categories. A score of 3 or less is deemed low risk, 3–5 medium risk and 6 or more is deemed high risk of an adverse outcome in the event of SARS-CoV-2 infection.^37^

The COVID-Age assessment tool also lists various risk factors, such as gender, ethnicity, age, obesity and other medical conditions. Each risk factor is allocated a score depending on the weighting of the individual risk factor. The total number is added to the patient's chronological age to give a score denoting ‘COVID age’.^38^ This algorithm-based tool was developed using data from a UK study of National Health Service (NHS) primary care records of more than 17 million adults examining which conditions or disorders predisposed to COVID-19-related death.^16^ Higher COVID-Age scores carry higher case fatality rates. A COVID-Age of 70 years corresponds to the equivalent case fatality rate of between 8 and 12.8% of an otherwise healthy 70-year-old man.

### Objectives

We set out to determine whether forensic psychiatric patients would be at greater than expected risk of adverse outcome in the event of infection with SARS-CoV-2, using two structured tools, the Covid-Risk assessment tool and the COVID-Age assessment tool.^37,38^ We considered that these patients would be a uniquely vulnerable group. We considered that vulnerability in the event of infection with SARS-CoV-2 would be greater than the risk expected for chronological age, particularly at the threshold level of risk for a healthy 70-year-old.

## Method

### Study design

This is a naturalistic cross-sectional observational study of a complete cohort of patients of a national forensic mental health service hospital for a population of 4.9 million in the Republic of Ireland. Demographic data and data pertaining to medical diagnoses were obtained from the patients’ medical records. The hospital has an on-site general practitioner and primary care nurse specialists, and data pertaining to physical health diagnoses were cross-checked with the primary care team notes.

### Setting

The Central Mental Hospital Dublin is the site of Ireland's National Forensic Mental Health Service (NFMHS). Patients include those who are on remand or sentenced and transferred to hospital or who have been found not guilty by reason of insanity or those detained under the Mental Health Act 2001 and deemed to have exceeded the capacity of their community mental health teams. The hospital is separated into eight different units with varying levels of therapeutic security along a coherent pathway through secure care, based on individual risks and needs.^39^ Patients are admitted to the higher-security wards, and progress in a stepwise manner to the lower secure units, before discharge to community units and supported living in the community.

### Participants

All patients (*n* = 141) under the care of the NFMHS on 14 September 2020 were included in the study.

### Variables

All patients in the NFMHS have a 6-monthly primary care review by a general practitioner and primary care nurse specialist, as required by the Mental Health Commission (Ireland). The two scales, the COVID-Risk (BMA) scale and the COVID-Age (ALAMA) scale, were completed for all 141 patients of the NFMHS during September 2020, by a psychiatric registrar and primary care nurse using up-to-date primary care records. Using the structured tools, a COVID-Risk and COVID-Age scores were computed for each patient drawing on the primary care records.

For sensitivity analysis a score was calculated for the number of diagnosed physical health conditions used to calculate Covid-Risk and Covid-Age, each rated present ‘1’ or absent ‘0’. These were body mass index (BMI) ≥30, hypertension, any cardiac condition, any asthma, any chronic obstructive pulmonary disease, any diabetes, any chronic kidney disease, any malignancy, any liver disease, any other neurological disorder, any organ transplant, any spleen disease, any rheumatoid arthritis or systemic lupus erythematosus or psoriasis, any other immunological disorder or being prescribed any immunosuppressive therapy or treatment.

### Ethics

The study was approved by the Research Ethics and Effectiveness Committee of the National Forensic Mental Health Service as an urgent service need evaluation (approval number AUD/06102020/MD). No intervention or randomisation was involved, all data were anonymised, no burden was placed on patients, and all patients had the potential to benefit from improved care that might follow from the study. The authors assert that this work complies with the Helsinki Declaration of 1975 and 2008 as a whole.

### Statistical methods

Analysis of variance (ANOVA) and paired *t*-tests were used to compare mean biological ages, COVID-Age scores and COVID-Risk scores. Confidence intervals of proportions were calculated using Wilson's method. Data were analysed using SPSS version 26 for Windows.

## Results

### Participants

All patients of the service on 14 September 2020 were included, a total of 141; of these, 66.7% (*n* = 94) were in-patients and 33.3% (*n* = 47) were community patients, including those in the hospital (95, 67.4%) and those living in the community units (46, 32.6%). There were 124 males (87.9%) and 17 females (12.1%). Mean age was 45.5 years (median 44.1, s.d. = 11.4). The majority of patients were White (*n* = 132, 93.6%); only 9 (6.4%) were Black.

The most common primary psychiatric diagnosis was schizophrenia (*n* = 96, 68.1%), followed by schizoaffective disorder (*n* = 24, 17%) and psychotic depression (*n* = 4, 3.5%). The majority of the patients were detained having been found not guilty by reason of insanity (*n* = 98, 68.8%), with the remainder being transferred prisoners (*n* = 15, 10.7%), unfit to stand trial (*n* = 9, 6.4%), detained under civil Mental Health Act sections (*n* = 16, 11.3%) or wards of court (*n* = 4, 2.8%).

### Main results

Physical health comorbidities were very common. Of the total sample, 58.9% (*n* = 83) were obese, defined as a BMI ≥ 30, and 20.6% (*n* = 29) were overweight (BMI = 25–29); 19.9% (*n* = 28) had a diagnosis of hypertension and 32 (22.7%) had type II diabetes mellitus.

**Fig. 1:**
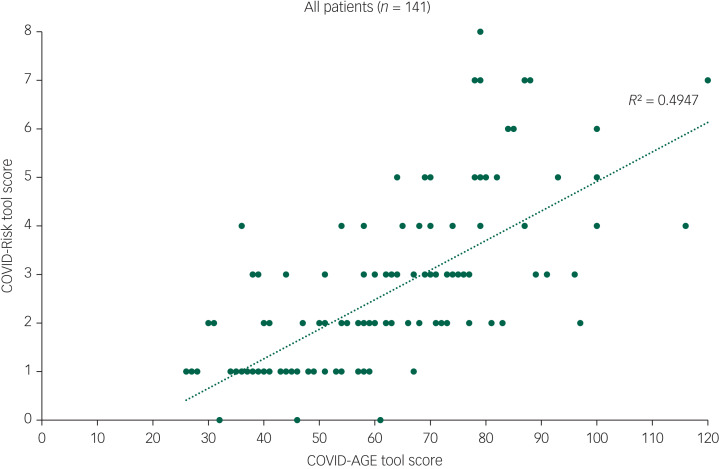
Association between COVID-Risk tool score and COVID-Age tool score: Spearman's *r* = 0.776, *P* < 0.001, *R*^2^ = 0.4947.

The mean chronological age was 45.5 years (median 44.1, s.d. = 11.4), whereas the mean COVID-Age score was 59.1 years (median 58.0, s.d. = 19.4) (paired *t*-test *t* = 10.347, *P* < 0.001) (Table 1). The mean COVID-Risk score was 2.4 (median 2, s.d. = 1.68). Figure 1 shows the positive association between COVID-Risk score and COVID-Age score (Spearman's *r* = 0.776, *P* = 0.000, *R*^2^ = 0.4947) with intersection at a COVID-Age score of 70 and COVID-Risk score of 3.

**Table 1:** Demographics, hospital units, COVID-Age and COVID-Risk scores

	*n* (%)	Chronological age, years: mean (median, s.d.)	COVID-Age score, mean (median, s.d.)	Paired-samples *t*-test (chronological age and COVID-Age), *t* (sig. 2-tailed)	Patients with COVID-Age ≥70, *n* (%, 95% CI)	COVID-Risk score, mean (median, s.d.)
Demographics						
Male	124 (87.9%)	45.1 (43.0, 11.86)	58.9 (58.0, 19.98)	9.566 (<0.001)	39 (31.5%, 23.9–40.1)	2.4 (2.0, 1.66)
Female	17 (12.1%)	48.8 (47.0, 7.35)	60.9 (59.0, 15.35)	4.155 (0.001)	4 (23.5%, 9.6–47.3)	2.4 (2.0, 1.90)
Hospital in-patients by location						
Women's in-patient unit	10 (7.1%)	47.0 (46.4, 7.56)	58.4 (59.5, 17.44)	2.585 (0.029)	2 (20%, 5.6–50.9)	2.2 (2.5, 1.87)
Male admissions unit	11 (7.8%)	35.7 (35.4, 10.60)	52.6 (44.0, 20.85)	3.099 (0.011)	2 (18.1%, 5.1–47.7)	2.0 (2.0, 1.10)
Medium secure unit 1	16 (11.3%)	38.8 (37.9, 7.09)	57.9 (52.5, 19.75)	4.530 (<0.001)	4 (25%, 10.2–49.5)	2.4 (2.0, 1.59)
Medium secure unit 2	16 (11.3%)	39.6 (39.4, 10.02)	50.0 (43.5, 18.82)	3.238 (0.006)	3 (18.8%, 6.6–43.0)	1.8 (1.5, 1.07)
High dependency unit	6 (4.3%)	52.0 (52.1, 11.50)	60.2 (53.5, 20.51)	1.867 (0.121)	2 (33.3%, 9.6–70.0)	3.2 (2.5, 2.40)
High dependency step-down unit	11 (7.8%)	46.6 (46.1, 8.23)	72.2 (71.0, 26.96)	3.383 (0.007)	6 (54.5%, 28.0–78.7)	2.9 (3.0, 1.70)
Rehabilitation unit	17 (12.1%)	53.0 (48.2, 13.49)	67.8 (69.0, 18.83)	4.245 (0.001)	8 (47.1%, 26.2–69.0)	3.1 (3.0, 1.95)
Intellectual disability unit	8 (5.7%)	37.8 (37.7, 8.51)	45.4 (42.0, 17.17)	1.815 (0.112)	1 (12.5%, 2.2–39.3)	1.6 (1.0, 1.41)
Summary						
Hospital in-patients	95 (67.4%)	43.6 (41.3, 11.5)	58.6 (58.0, 21.17)	8.774 (<0.001)	28 (29.5%, 21.2–39.3)	2.4 (2.0, 1.67)
Community patients	46 (32.6%)	49.5 (48.1, 10.46)	60.3 (58.5, 15.66)	5.596 (<0.001)	12 (26.1%, 15.5–40.3)	2.5 (2.0, 1.75)
Total	141 (100%)	45.5 (44.1, 11.46)	59.1 (58.0, 19.45)	10.347 (<0.001)	40 (30.5%, 23.5–38.5)	2.4 (2.0, 1.68)

sig., significant.

Over one-third of the patient group (*n* = 54; 38%) rated as medium or high risk on the COVID-Risk assessment tool; 33% (*n* = 47) had a COVID-Age score of 50–70 years, 17% (*n* = 24) had a COVID-Age of 71–80 and 14% (*n* = 19) had a COVID-Age >80. Therefore, a total of 30.5% of the total patient group had a COVID-Age over 70 years. The main contributors to increased COVID-Age and high COVID-Risk scores were increased BMI, followed by cardiac illness, including hypertension and heart failure.

There was no difference between male and female patients, despite female gender conveying a protective effect equivalent to minus 5 years in COVID-Age. Hospital and community patients also did not differ significantly on either measure.

In the complete sample, three patients (2.1%) had a chronological age ≥70 years, compared with 43 (30.5%) with a COVID-Age ≥70 (χ^2^ = 6.99, d.f. = 1, *P* = 0.008, Fisher's exact test *P* = 0.027).

#### Risks identified on COVID-Risk and COVID-Age tools and placement in the hospital

The NFMHS consists of high, medium and low secure units, all on one site with follow-up community placement units for conditionally discharged patients. Individual patients are admitted to higher secure units within the hospital, and progress along a therapeutic pathway through secure care onto medium secure and finally rehabilitation units. A small number require high dependency care, typically to manage treatment-resistant psychoses with ongoing violence.

**Fig. 2:**
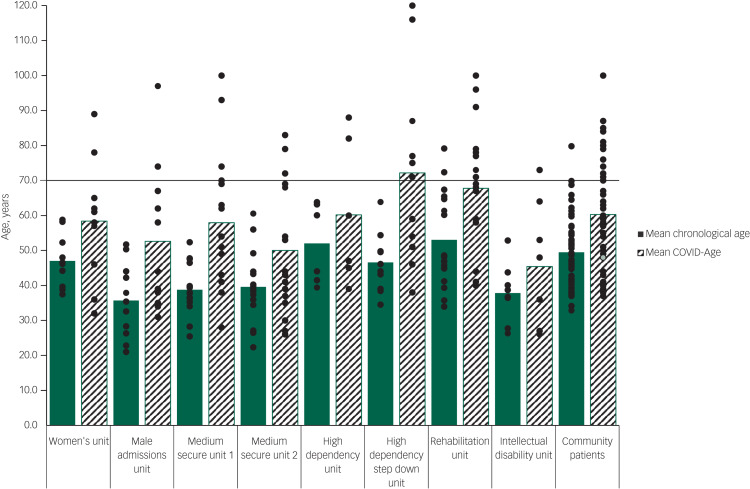
Mean chronological age and mean COVID-Age score in each hospital unit.

The differences between mean chronological age and mean COVID age along the care pathway are shown in Table 1 and Fig. 2. Mean COVID-Age was significantly higher than mean chronological age across every unit within the hospital (Table 1); this included male patients, female patients and patients on the intellectual disability ward. Patients with a COVID-Age ≥70 are the high-risk group as identified by the COVID-Age tool. We found that this group was dispersed across the hospital, with patients deemed at high risk of adverse outcome in the event of SARS-CoV-2 infection on every ward.

### Sensitivity analysis

The calculated mean number of physical health diagnoses was 1.57 (s.d. = 1.27). There was no difference in mean number between men (1.53, s.d. = 1.27) and women (1.82, s.d. = 1.38) (ANOVA *F* = 0.78, *P* = 0.38). There was also no difference between Black patients (*n* = 9) and White patients (*n* = 132): White, 1.56, (s.d. = 1.29); Black, 1.67 (s.d. = 1.00) (ANOVA *F* = 0.06, *P* = 0.811).

For sensitivity analysis a score was calculated for the number of physical health diagnoses used to calculate COVID-Risk and COVID-Age scores, each rated present ‘1’ or absent ‘0’. These were BMI ≥ 30 (*n* = 83, 58.9%), hypertension (*n* = 28, 19.9%), any cardiac condition (*n* = 6, 4.3%), any asthma (*n* = 20, 14.2%), any chronic obstructive pulmonary disease (*n* = 7, 5.0%), any diabetes (*n* = 32, 22.7%), any chronic kidney disease (*n* = 8, 5.7%), any malignancy (*n* = 7, 5.0%), any liver disease (*n* = 8, 5.7%), any other neurological disorder (*n* = 5, 3.5%), any organ transplant (0), any spleen disease (*n* = 1, 0.7%), any rheumatoid arthritis or systemic lupus erythematosus or psoriasis (*n* = 13, 9.2%), any other immunological disorder (0) or being prescribed any immunosuppressive therapy or treatment (*n* = 3, 2.1%).

Twenty-eight patients (19.9%) had no comorbid physical health diagnoses relevant to risk as rated by these tools; 51 (36.2%) had one such diagnosis; 34 (24.1%) had two such diagnoses; 15 (10.6%) had three physical health diagnoses; 8 (5.7%) had four such diagnoses; and 5 patients (3.5%) had five physical health diagnoses relevant to risk as rated on these tools. Biological age increased significantly with increasing numbers of physical health diagnoses relevant to risk (ANOVA *F* = 3.08, *P* = 0.012). COVID-Age also increased significantly with an increasing number of physical health diagnoses (ANOVA *F* = 30.839, *P* < 0.001), as did COVID-Risk (ANOVA *F* = 18.230, *P* < 0.001).

Ninety-four patients were hospital residents and 47 were in the community. Community patients were significantly older than hospital patients (hospital mean age 43.64 years, s.d. = 11.53; community mean age 49.34 years, s.d. = 10.38; ANOVA *F* = 8.175, *P* = 0.005). However, they did not differ in COVID-Age (hospital mean COVID-Age 58.64, s.d. = 21.17; community mean COVID-Age 60.02, s.d. = 15.59; ANOVA *F* = 0.158, *P* = 0.692). There was also no significant difference in COVID-Risk scores between the hospital and community groups (hospital mean COVID-Risk 2.39, s.d. = 1.66; community mean 2.49, s.d. = 1.74; ANOVA *F* = 0.101, *P* = 0.751). The mean number of physical diagnoses relevant to risk was 1.66 (s.d. = 1.29) in the hospital sample and 1.38 (s.d. = 1.23) in the community sample (ANOVA *F* = 1.473, *P* = 0.227). It is of note that there were no differences in the mean COVID-Risk and COVID-Age scores between the hospital and community patient groups, despite the fact that the community group had an older chronological age and those in the community would have greater access to cigarettes and food choices that might predispose patients to obesity.

## Discussion

Secure forensic mental health services provide care and treatment to mentally disordered offenders with high levels of treatment-resistant mental illness and high levels of violence.^24^ However, increasingly the links between major mental disorder, especially treatment-resistant schizophrenia, and significant medical comorbidities, particularly obesity, are emerging.^40^ Both obesity and schizophrenia are hypothesised to be associated with inflammatory processes and this could render patients in secure forensic settings, who typically suffer from highly treatment-resistant mental illnesses, uniquely vulnerable in terms of their physical health.^40^ In this study we found significant differences between patients' biological age and COVID-Age score in a national cohort of patients in a forensic mental health service, both in hospital and in the community. This cohort is highly selected from among all the mental health patients in a population of 4.9 million. Although high rates of medical comorbidities are typically found in individuals with treatment-resistant psychoses, the fact that almost one-third of the population of patients of a national forensic mental health service met COVID-Age criteria for high risk, with a COVID-Age ≥70 years, was unexpected and concerning. This patient group are, in our view, uniquely vulnerable to adverse outcome in the event of infection with SARS-CoV-2.

In terms of managing this risk of adverse outcomes in the event of SARS-CoV-2 infection, the typical strategy of services would be to issue guidance to these individuals to self-isolate as much as possible. Governments have advocated population-based cocooning and self-isolation based on a chronological age of ≥70 years, but this may not be sufficient in this patient group. Given that the patient group identified as high risk using both tools were distributed across all wards and units of the hospital, it follows that hospital-wide measures would be needed to best mitigate the identified risk. Therefore, measures such as limiting visitors on site and reducing the need for multidisciplinary teams to move between units by basing teams on individual wards may need to be considered in forensic settings.^1^

### Limitations

Both the COVID-Risk assessment tool and the COVID-Age assessment tool were developed for use in occupational health assessments of healthcare workers. We used these scales in this project because, to date, there are no validated scales available that were developed specifically for patients. Second, this project consisted of a chart review only. Where patients were being investigated for medical diagnoses, if these diagnoses were not formally made at the time of the study they were not included, so that some scores may have been an underestimation of the true risk. Data were abstracted by the psychiatric registrar for the patients’ care teams and the primary care nurse specialist working together. As the primary care nurse specialist works across all patient groups in the forensic service, this limited problems relating to interrater reliability.

A difference in our sample compared with the UK's secure forensic patient groups is that a small proportion of our group, only 6.4%, were from a Black and minority ethnic background. We are of the view that in UK secure forensic mental health services this group would make up a significantly larger proportion of the patient cohort.^41^ This is also likely to be the case in forensic psychiatric populations in Canada, Australia, the USA and other jurisdictions. Given the risks of adverse outcome in the event of COVID-19 infection for this vulnerable group, we are of the view that this would increase the risks further in those settings.

### Interpretation and generalisability

Although not yet established in a prospective study in this patient group, we believe that these risk tools have utility in identifying the extent of those at highest level of risk, among a uniquely medically vulnerable patient group. Such tools guide clinicians but do not bind them. We consider that patients in secure forensic psychiatric services are at high risk for adverse outcomes in the event of SARS-CoV-2 infection. Because all in-patients in a secure setting are involuntarily detained under mental health legislation, there is an added duty of care to identify those at high risk and manage the risk appropriately. Population-based cocooning and self-isolating guidance based on chronological age may not be sufficient in this population.

There are approximately 6000 patients in secure forensic settings in high and medium secure hospitals throughout the UK and the Republic of Ireland. There are many more in secure forensic hospitals across Europe, North America and elsewhere. We believe that the risks identified in this group are very likely to be generalisable to those hospitals. Their patients have similar psychiatric diagnoses and physical health comorbidities. We believe that these findings draw attention to the urgent need to focus on physical health and risks for patients in secure forensic hospitals. We believe that these findings are also likely to be relevant to patients with severe and enduring mental illness in general psychiatry settings. Research on risk mitigation strategies concerning the vulnerability of this group to adverse outcomes in the event of infection with SARS-CoV-2 is urgently required. This must focus on short-term as well as medium-term strategies. Conventional approaches emphasise medium-term interventions such as smoking cessation, improving diet and management of cardiovascular risk factors. Short-term strategies at present emphasise prevention of transmission of infection in secure hospital settings but should also include patient and staff education and motivation work emphasising the need for vaccine uptake.^42^ We believe that this is relevant to decisions regarding the prioritisation of vulnerable groups for accessing COVID-19 vaccination programmes.^43^

## Data Availability

Owing to the sensitive nature of the data-set, access to data will only be granted on a case-by-case basis.
